# Anisotropy component of DTI reveals long-term neuroinflammation following repetitive mild traumatic brain injury in rats

**DOI:** 10.1186/s41747-024-00490-w

**Published:** 2024-07-24

**Authors:** Ching Cheng, Chia-Feng Lu, Bao-Yu Hsieh, Shu-Hui Huang, Yu-Chieh Jill Kao

**Affiliations:** 1 Department of Biomedical Imaging and Radiological Sciences, National Yang Ming Chiao Tung University, Taipei, Taiwan; 2 Department of Medical Imaging and Radiological Sciences, College of Medicine, Chang-Gung University, Taoyuan, Taiwan; 3 Department of Medical Imaging and Intervention, Chang Gung Memorial Hospital at Linkou, Taoyuan, Taiwan

**Keywords:** Brain concussion, Diffusions tensor imaging, Neuroinflammatory diseases, Rats, White matter

## Abstract

**Background:**

This study aimed to investigate the long-term effects of repetitive mild traumatic brain injury (rmTBI) with varying inter-injury intervals by measuring diffusion tensor metrics, including mean diffusivity (MD), fractional anisotropy (FA), and diffusion magnitude (L) and pure anisotropy (q).

**Methods:**

Eighteen rats were randomly divided into three groups: short-interval rmTBI (*n* = 6), long-interval rmTBI (*n* = 6), and sham controls (*n* = 6). MD, FA, *L*, and *q* values were analyzed from longitudinal diffusion tensor imaging at days 50 and 90 after rmTBI. Immunohistochemical staining against neurons, astrocytes, microglia, and myelin was performed. Analysis of variance, Pearson correlation coefficient, and simple linear regression model were used.

**Results:**

At day 50 post-rmTBI, lower cortical FA and *q* values were shown in the short-interval group (*p* ≤ 0.038). In contrast, higher FA and *q* values were shown for the long-interval group (*p* ≤ 0.039) in the corpus callosum. In the ipsilesional external capsule and internal capsule, no significant changes were found in FA, while lower *L* and *q* values were shown in the short-interval group (*p* ≤ 0.028) at day 90. The *q* values in the external capsule and internal capsule were negatively correlated with the number of microglial cells and the total number of astroglial cells (*p* ≤ 0.035).

**Conclusion:**

Tensor scalar measurements, such as *L* and *q* values, are sensitive to exacerbated chronic injury induced by rmTBI with shorter inter-injury intervals and reflect long-term astrogliosis induced by the cumulative injury.

**Relevance statement:**

Tensor scalar measurements, including *L* and *q* values, are potential DTI metrics for detecting long-term and subtle injury following rmTBI; in particular, *q* values may be used for quantifying remote white matter (WM) changes following rmTBI.

**Key Points:**

- The alteration of L and q values was demonstrated after chronic repetitive mild traumatic brain injury.
- Changing q values were observed in the impact site and remote WM.
- The lower q values in the remote WM were associated with astrogliosis.

**Graphical Abstract:**

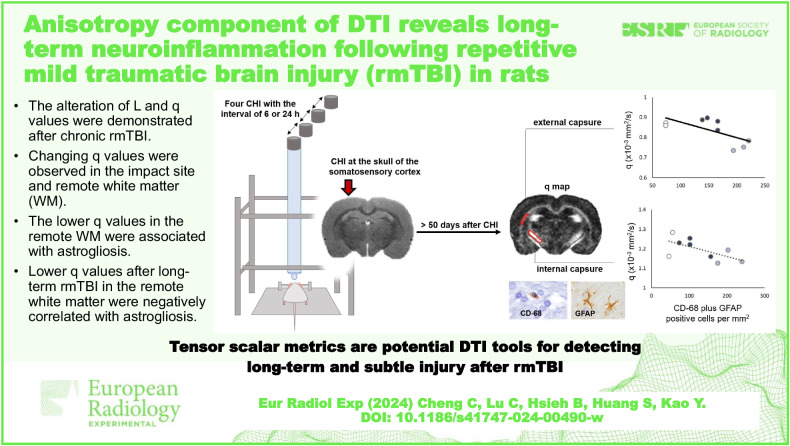

## Background

Traumatic brain injury affects a significant number of individuals worldwide, with over 69 million people experiencing TBI each year [1]. Among these cases, mild traumatic brain injury (mTBI) stands as the predominant variant, accounting for nearly 85% of the overall TBI occurrences [2]. Repetitive mTBI (rmTBI) has gained significant attention, particularly concerning the population such as football players and military veterans, who often report persistent post-concussive symptoms such as headaches, concentration problems, memory impairment, depressive mood disorders, and balance difficulties [1, 3–6]. The cumulative effects of consecutive injuries increase susceptibility to subsequent mTBI and induce long-lasting neuroinflammation [7, 8]. In addition to the number of prior mTBI, the concept of a period of vulnerability after repeated brain injury has been postulated, suggesting that ensuing mTBI may manifest the outcome before the brain recovers from the previous injury [9, 10].

Negative findings in conventional magnetic resonance imaging (MRI) or computed tomography have always been reported after mTBI, specifically uncomplicated mTBI, posing challenges to effective medical decision-making and putative injury management [11]. Diffusion MRI has been proposed to reflect the microstructural integrity disrupted by mTBI and predict clinical patient outcomes. While the decreased apparent diffusion coefficient was not found until white matter (WM) lesions were presented [12], changes in fractional anisotropy (FA) after mTBI reflect the loss of microstructural integrity induced by injury [13, 14]. Recently, the alteration in scalar measurements of diffusion tensor transformation, including the *q* value, the anisotropic component, and the *L* value, the total value of diffusion tensor, has been reported in various pathological conditions including but not limited to gliomas, glioblastoma multiforme, stroke, intracranial epidermis, normal pressure hydrocephalus, and age-related degeneration [15–25]. With the combination of changes among diffusion scalar measurements, different severity of WM damage affected by glioblastoma was reported, which was further utilized to categorize the brain tumors [23, 26]. As heterogeneous injury in WM, after rmTBI was anticipated, we thus attempted to delineate the severity and progression of rmTBI-induced changes in diffusion tensor imaging (DTI) metrics by using diffusion scalar measurements.

In this study, we used a closed-head injury (CHI) rat model imitating clinical uncomplicated mTBI [27] to replicate rmTBI with different inter-injury intervals, addressing the effect of rmTBI history. In contrast to the prevalent studies assessing image biomarkers in the acute phase after injury [28–30], we focused on diffusion MRI in the long-term phase after rmTBI. We hypothesize that more severe injury by shortening inter-injury intervals may exacerbate the chronic injury after rmTBI, subsequently yielding discernible alterations in diffusion tensor scalar measurements as surrogate markers of cumulative injury effects.

## Methods

All surgical procedures and experiments were approved by the local Institute of Animal Care and Utilization Committee (NYCU IACUC 1090518, 5/26/2020, Taipei, Taiwan). Adult male Sprague Dawley rats aged 10 to 12 weeks and weighing 250 to 400 g were used. Rats were housed with a 12 h:12 h light/dark cycle, and food and water were available ad libitum.

### Closed-head injury model

Eighteen animals were randomly assigned to three experimental groups, including the short-interval rmTBI group (*n* = 6), the long-interval rmTBI group (*n* = 6), and the sham control group (*n* = 6). Repetitive mTBI was induced according to the CHI model, which is a modified weight-drop injury model [27]. Briefly, a midline incision was performed on the scalp, and a circular stainless steel helmet was cemented over the skull on top of the left sensorimotor cortex (1.5 mm posterior and 2.5 mm lateral to the bregma). A 600-g weight was dropped from a height of 1 m through a stainless steel tube to the secured impactor with a round tip aimed at the metal helmet. Animals received four impacts with a 6-h and a 24-h interval in the short and long-interval rmTBI group, respectively [9]. Animals in the sham control group underwent procedures identical to those of the rmTBI rats without receiving actual impacts.

### MRI protocol

Longitudinal MRI was acquired using a 7-T PharmaScan scanner (Bruker BioSpin, GmbH, Rheinstetten, DE) at day 50 and day 90 after sham or rmTBI surgery. A volume coil was used for radiofrequency excitation, and an array coil was used for signal reception. Rats were anesthetized under ~1.2% isoflurane. After initial localization scans, T2-weighted images and DTI were acquired (Supplementary Table S1). During the scan, body temperature was maintained with a hot water circulation system, and heart rate, arterial pulse extension, and oxygen saturation were monitored with a pulse oximeter (MouseOx®, Starr Life Sciences Corp., Oakmont, USA).

### Image analysis

**Fig. 1:**
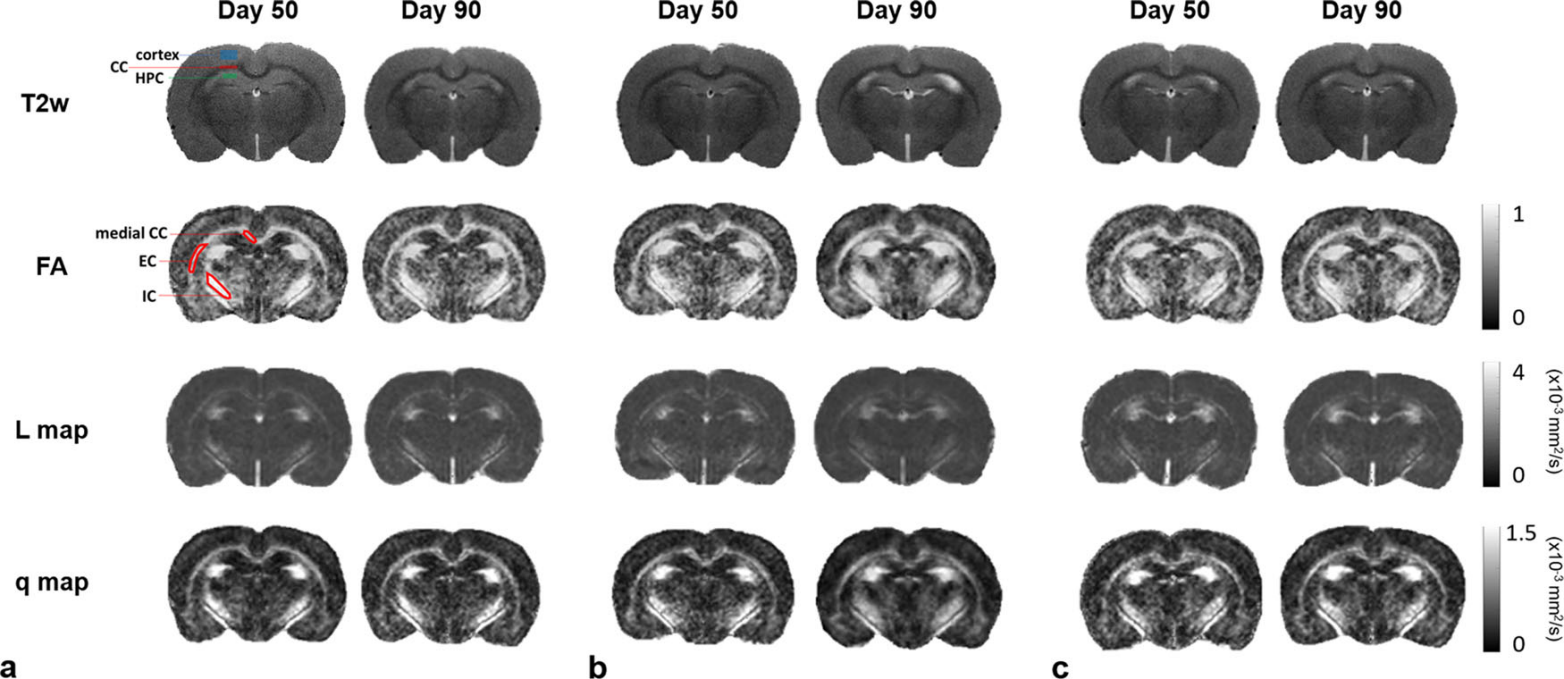
No contusion or edema was observed in the brain at day 50 and day 90 of a sham rat (**a**) and after rmTBI, regardless of the short-interval (**b**) or long-interval (**c**) groups. The regions labeled in the T2w images and FA map for sham group rats were the regions of interest used for the quantitative analysis. EC, External capsule; CC, Corpus callosum; FA, Fractional anisotropy; HPC, Hippocampus; IC, Internal capsule; T2w, T2-weighted images

Image analysis, including skull stripping and motion correction/coregistration across time points and subjects, was performed using Statistical Parametric Mapping software (http://www.fil.ion.ucl.ac.uk/spm/software/spm12) and a custom Matlab script (MathWorks, Natick, Massachusetts, USA), published previously [27, 31, 32]. DTI metrics, including mean diffusivity (MD), fraction anisotropy (FA), pure anisotropy (*L*), and diffusion magnitude (*q*) value, were generated from each subject. The scalar measures *q* and *L* values were calculated based on the following equation [19, 33]:

$$FA=\sqrt{\frac{3}{2}}\frac{\sqrt{{({\lambda }_{1}-\bar{\lambda })}^{2}+{({\lambda }_{2}-\bar{\lambda })}^{2}+{({\lambda }_{2}-\bar{\lambda })}^{2}}}{\sqrt{{\lambda }_{1}^{2}+{\lambda }_{2}^{2}+{\lambda }_{3}^{2}}}=\sqrt{\frac{3}{2}}\frac{q}{L}$$

where λ_*i*_ were the eigenvalues of the diffusion tensor matrix, and $\bar{\lambda }$ was the mean diffusion. Region of interests (ROIs), including the cortex, corpus callosum (CC), hippocampus under the impact position, and the medial CC, external capsule (EC), internal capsule (IC), and the remote WM were delineated based on the rat brain atlas [34] by two independent operators with ten and three years of experience in rat brain segmentation, blinded to the experimental groups (Fig. 1a, Supplementary Table S2) [27]. Mean MD, FA, *L*, and *q* values were calculated for all the ROIs.

### Immunohistochemistry

At day 90 post-rmTBI or sham surgery, a subgroup of animals of the short-interval rmTBI group (*n* = 3), of the long-interval rmTBI group (*n* = 4), and of the sham control group (*n* = 4) in the) were anesthetized and transcardially perfused with saline and formaldehyde to harvest the brain tissue [27]. Immunohistochemical stains for neuronal nuclear antigen (NeuN), glial fibrillary acidic protein (GFAP), cluster of differentiation 68 (CD-68), and myelin basic protein (Supplementary Table S3) were performed to assess neuronal loss, accumulation of astrocytes and microglia, and axonal injury, respectively, on coronal sections. The immunohistochemistry data were viewed using a BX63 light microscope slide scanner (Olympus, Shinjuku, Japan). ROIs (Supplementary Fig. S3a–d) were traced to quantify the number of activated astrocytes, microglia, and neurons by using Image J software (National Institutes of Health, Bethesda, Maryland, USA).

### Statistical analysis

Statistical analyses were performed using SPSS software (IBM, Armonk, New York). In each ROI, the differences in certain DTI metrics among groups of animals across longitudinal MRI acquisitions were determined by the repeated measures of ANOVA (rmANOVA), where the between-subjects factor was “groups” and the within-subjects factor was “temporal change”. When differences were found between groups, a post hoc test was performed using Bonferroni corrections (type I error = 0.05/3). The differences in each group in NeuN, GFAP, and CD-68 expressions at day 90 post-rmTBI were determined by one-way ANOVA. A post hoc test was performed using the Tukey honestly significant difference test as all the datasets passed the homogeneity test. The relationship between the tensor scalar measurements and the immunohistochemical results was determined by the Pearson correlation coefficient and simple linear regression model. Statistical significance was defined as *p* < 0.05 (SPSS adjusted).

## Results

Compared with the sham group and the contralesional side of the brain, no focal contusion nor tissue loss was observed in the ipsilesional hemisphere at both day 50 and day 90 after rmTBI regardless of the inter-injury intervals (Fig. 1). There were no significant changes in MD underneath the impact region (Supplementary Fig. S2a) after rmTBI regardless of the inter-injury intervals. In addition, no significant changes between days 50 and 90 were observed for all DTI metrics.

### Longitudinal analysis of DTI metrics underneath the impact site

**Fig. 2:**
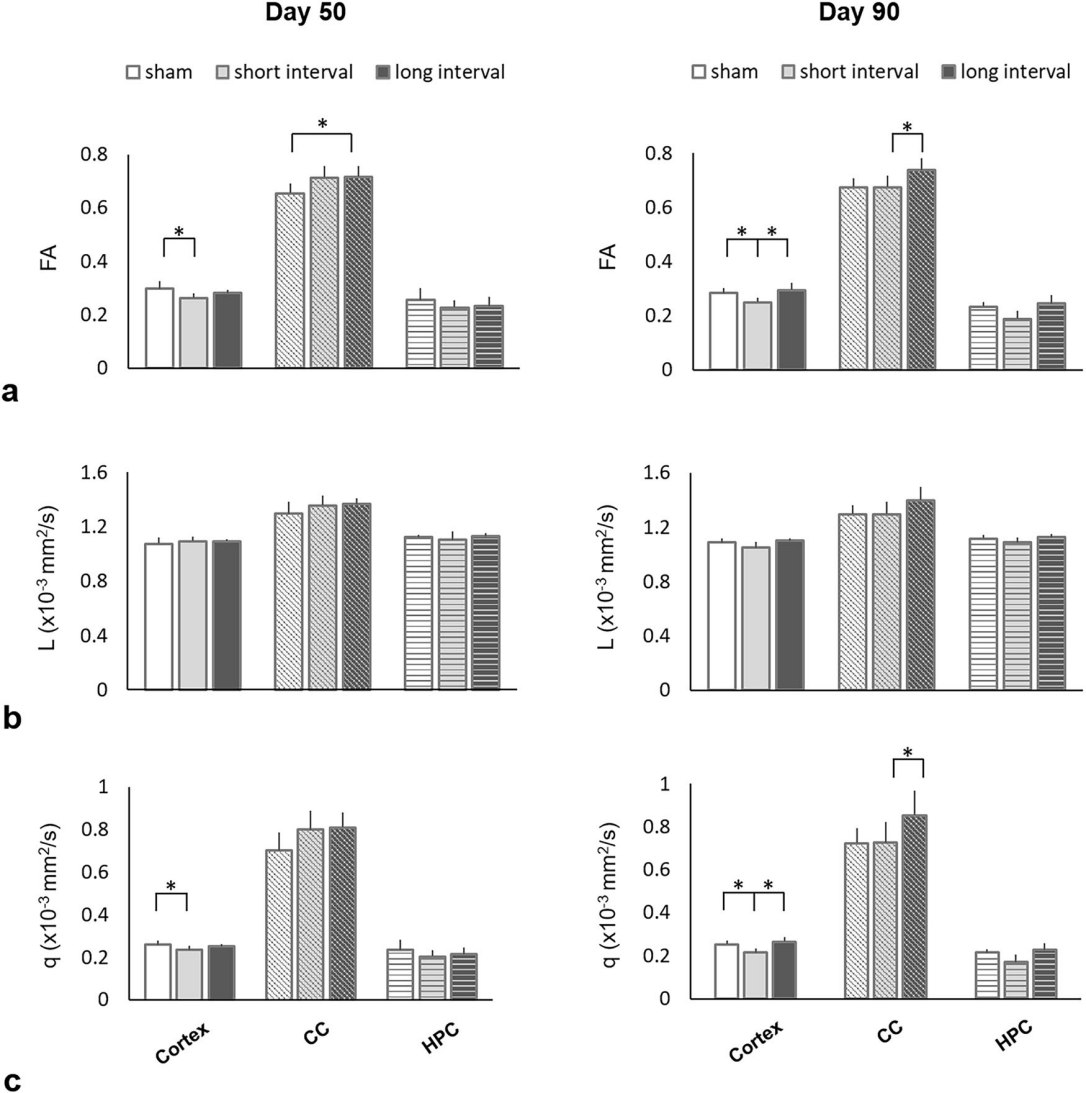
Mean fractional anisotropy (**a**), L (**b**), and q (**c**) values from the cortex, corpus callosum (CC), and hippocampus (HPC) under the impact site at day 50 and day 90 in the sham group and after rmTBI. * indicates significant differences between groups (*p* ≤ 0.039)

In the cortex, a significantly lower FA in the short-interval group was shown compared with that in the sham group at day 50 after rmTBI (*p* = 0.035). The lower FA sustained at day 90 showed a significant difference when compared to both the sham and the long-interval group (*p* = 0.004 and *p* = 0.008, respectively) (Fig. 2a). In the CC, a significantly higher FA was observed in the long-interval group compared with that in the sham group at day 50 (*p* = 0.039) and compared with that in the short-interval group at day 90 (*p* = 0.032). No significant difference was found in L underneath the impact region after rmTBI regardless of the inter-injury intervals (Fig. 2b). In the cortex, significantly lower *q* values in the short-interval group were shown compared with that in the sham group at day 50 after rmTBI (*p* = 0.038) (Fig. 2c). The lower *q* values sustained at day 90 showed significant differences with both the sham and the long-interval group (*p* = 0.001 and *p* = 0.002, respectively). In the CC, significantly higher *q* values were observed in the long-interval group than in the short-interval group at day 90 (*p* = 0.035).

### Longitudinal analysis of DTI metrics in the remote WM

**Fig. 3:**
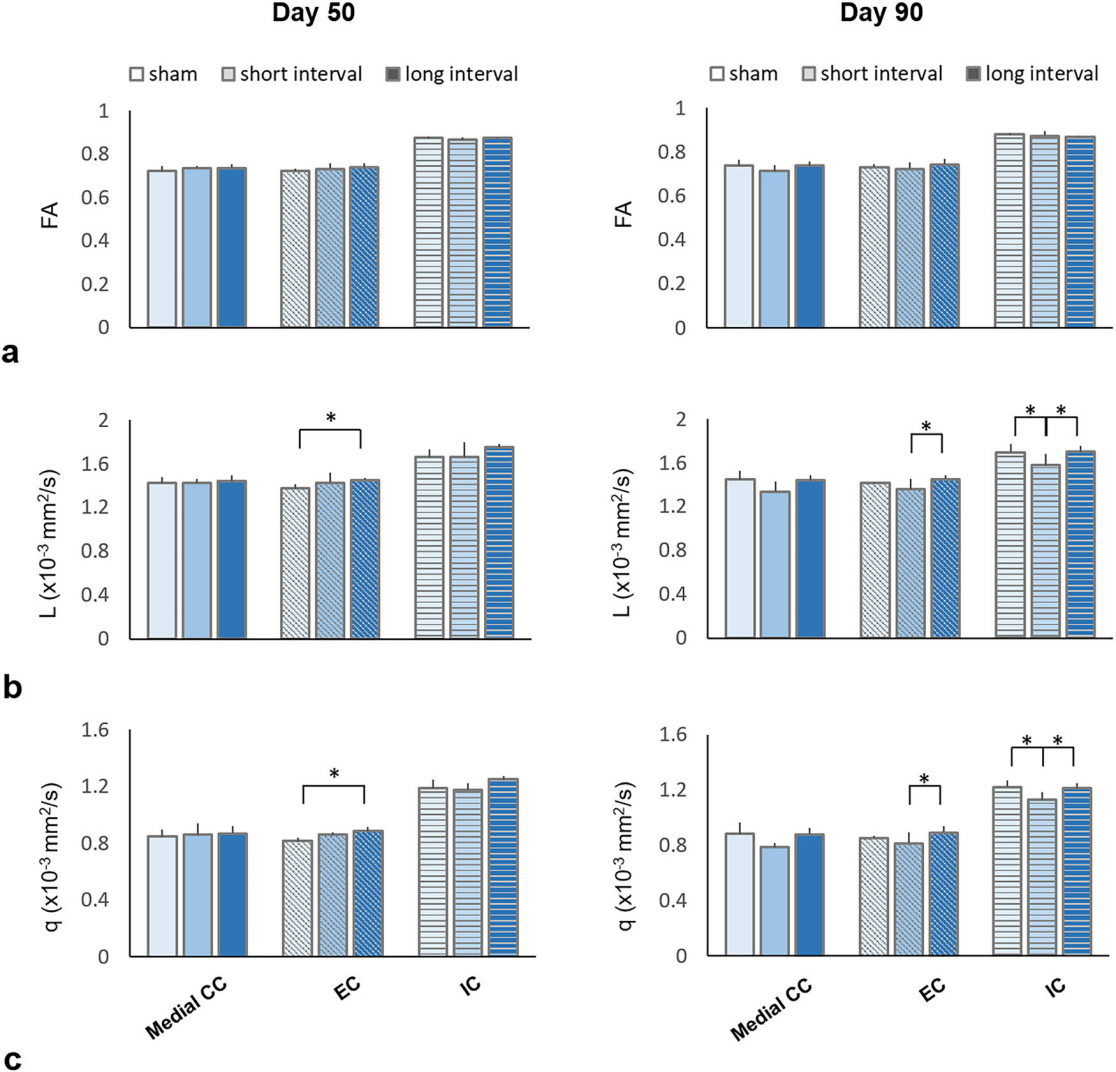
Mean fractional anisotropy (**a**), L (**b**), and q (**c**) values from the medial corpus callosum (medial CC), external capsule (EC), and internal capsule (IC) at day 50 and day 90 in the sham group and after mild traumatic brain injury. * indicates significant differences between groups (*p* ≤ 0.028)

In the remote WM, while no significant difference was found in FA after rmTBI regardless of the inter-injury intervals (Fig. 3a), changes in *L* and *q* values were observed after rmTBI. Significantly higher *L* values in the EC were shown in the long-interval group compared with the sham group at day 50 (*p* = 0.026) (Fig. 3b). The *L* values in the EC remained high at day 90 showing a significant difference with the short-interval group (*p* = 0.003). In the IC, significantly lower *L* values were observed in the short-interval group compared with both the sham and long-interval groups at day 90 after rmTBI (*p* = 0.028 and *p* = 0.002, respectively) (Fig. 3b). Also, significantly higher *q* values in the EC were shown in the long-interval group compared with the sham group at day 50 (*p* = 0.027) (Fig. 3c). The *L* values in the EC remained high at day 90 showing a significant difference with the short-interval group (*p* = 0.009). In the IC, significantly lower *q* values were observed in the short-interval group compared with both the sham and long-interval groups at day 90 after rmTBI (*p* = 0.005 and *p* = 0.001, respectively) (Fig. 3c).

### Immunohistology underneath the impact site

**Fig. 4:**
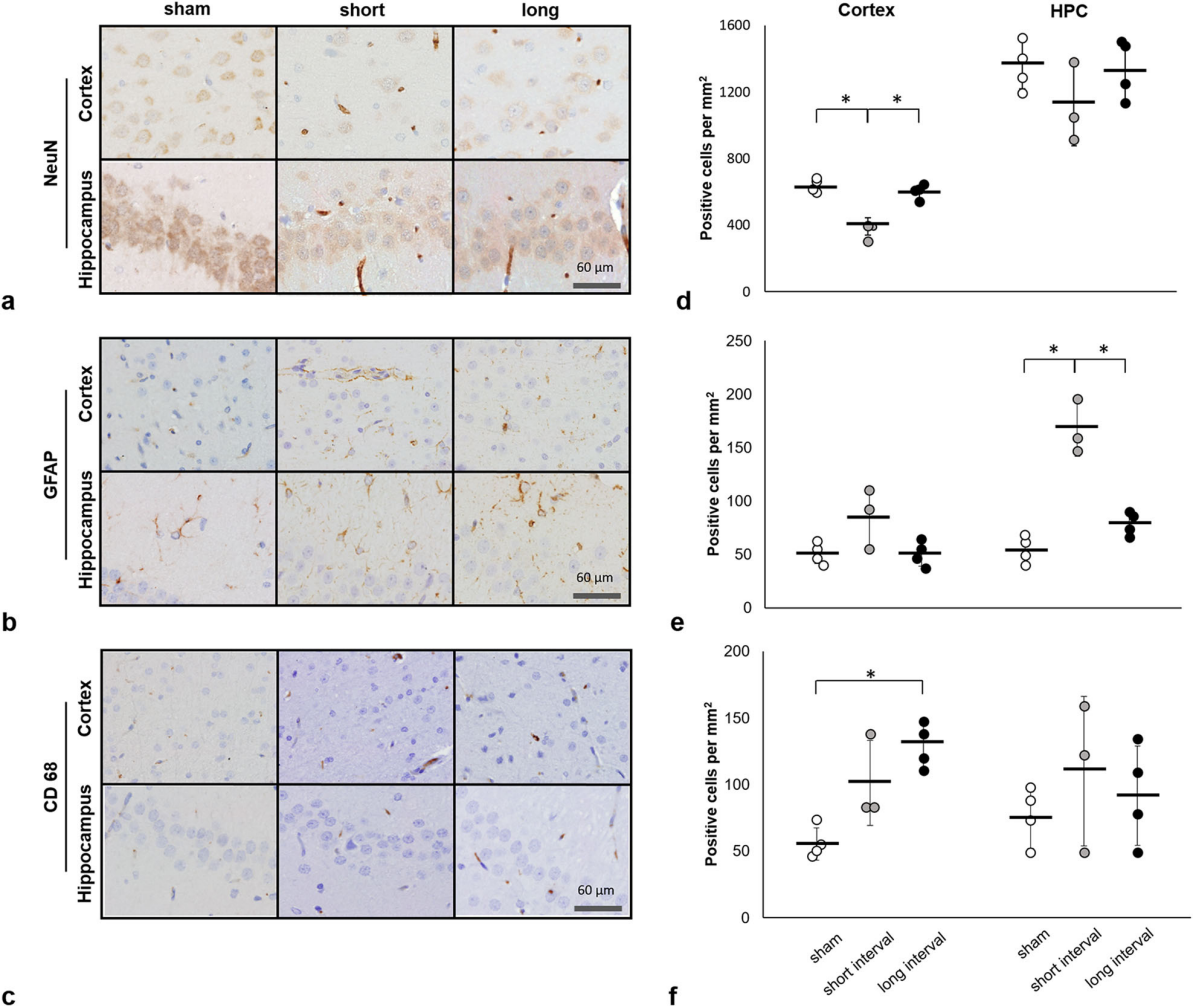
Neuronal nuclear antigen (NeuN)-positive (**a**), glial fibrillary acidic protein (GFAP)-positive (**b**), and Cluster of differentiation 68 (CD-68)-positive (**c**) staining at 90 days after mTBI. Quantification expression of NeuN (**d**), GFAP (**e**), and CD-68-positive (**f**) cells in the cortex and hippocampus (HPC). * indicates significant differences between groups (*p* ≤ 0.016)

At day 90 after rmTBI, in the cortex, significant loss of neurons was observed in the short-interval group compared with the sham and the long-interval group (*p* < 0.001 for both) (Fig. 4a, d). A significant increase of CD-68-labeled cells was observed in the long-interval group compared with the sham group (*p* = 0.004) (Fig. 4c, f). In the hippocampus, a significant increase in GFAP-labeled cells was observed in the short-interval group compared with the sham and the long-interval group (*p* = 0.007 and *p* = 0.016, respectively) (Fig. 4b, e).

### Immunohistology in the remote WM

**Fig. 5:**
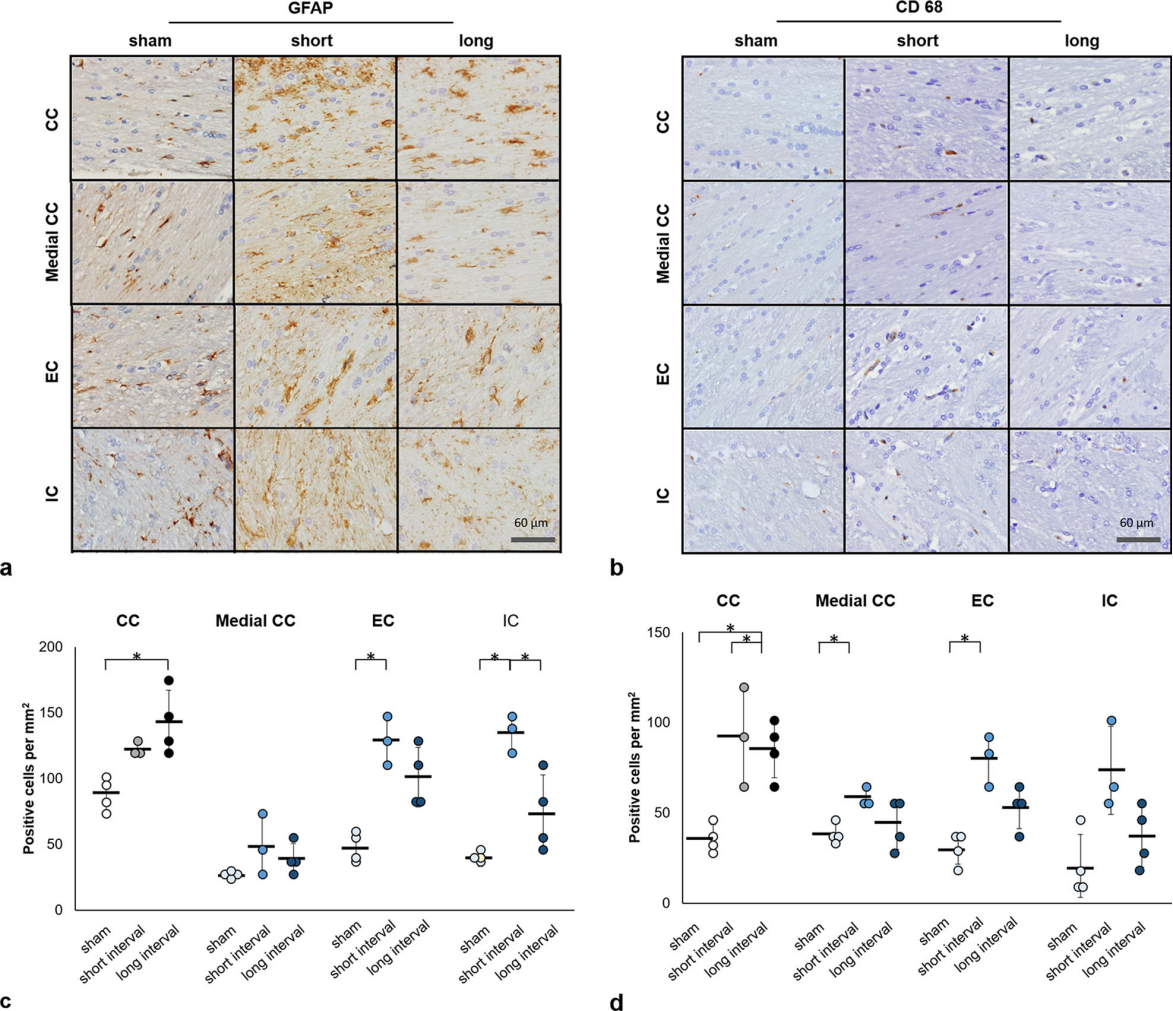
GFAP-positive (**a**) and CD-68-positive (**b**) staining in the white matter (WM) 90 days after mTBI. Quantification expression of GFAP (**c**) and CD-68-positive (**d**) cells in the corpus callosum (CC) underneath the impact site and the remote WM, including the medial corpus callosum (medical CC), external capsule (EC), and internal capsule (IC). * indicates significant differences between groups (*p* ≤ 0.037)

While no descent loss of myelin basic protein staining in WM was found among the three groups (Supplementary Fig. S3e), astroglial, and microglial expression were observed at day 90 after rmTBI (Fig. 5a, b). In the CC underneath the impact region, a significant increase of GFAP-labeled cells was observed in the long-interval group compared with the sham group (*p* = 0.037) (Fig. 5c). Significant increase of CD-68-labeled cells was observed in both rmTBI groups compared with the sham group (*p* = 0.020 and *p* = 0.027, respectively) (Fig. 5d). In the medial CC, significant increase of CD-68-labeled cells was observed in the short-interval group compared with the sham group (*p* = 0.033) (Fig. 5d). In the EC, significant increase of GFAP-labeled cells and CD-68-labeled cells was observed in the short-interval group compared with the sham group (*p* = 0.009 and *p* = 0.004, respectively) (Fig. 5c, d). In the IC, a significant increase of GFAP-labeled cells was observed in the short-interval group compared with the other groups (*p* = 0.009 and *p* = 0.026 in the sham and long-interval group, respectively) (Fig. 5c).

### Correlation between diffusion tensor scalar metrics and immunohistology in the remote WM

**Fig. 6:**
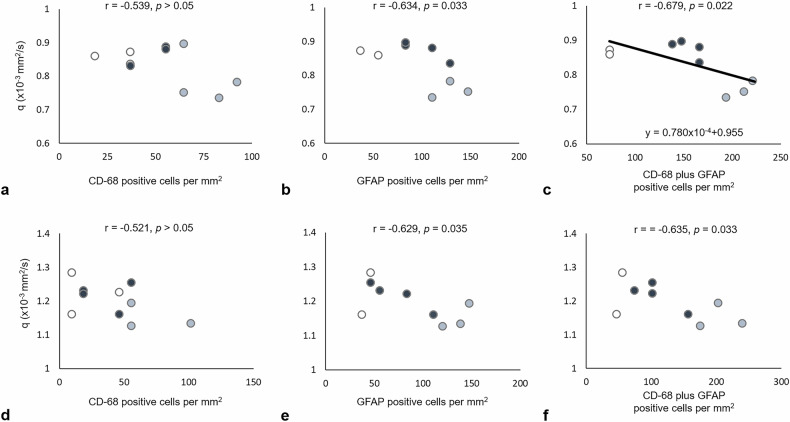
Correlation of the anisotropic component (*q* value) of diffusion tensor imaging in the external capsule (**a**, **b**, **c**) and internal capsule (**d**, **e**, **f**) at day 90 after mTBI with the corresponding immunohistochemistry-positive cells. The solid black lines in **c** represent the estimated linear regression line. The white, gray, and black dots denoted data from the sham, short-interval, and long-interval groups

Since significant changes in *L* and *q* values were found in the EC and IC at 90 days after rmTBI, correlation analysis between the scalar measurements and the number of astroglial cells stained against CD-68 and GFAP was performed. There was no significant correlation between the *L* value and the number of astroglial cells in the remote WM (Supplementary Fig. 3). While no relationship was observed between *q* value and the number of CD-68-positive cells, *q* values in both the EC and IC were negatively correlated with the number of GFAP-positive cells, with *r* values of -0.63 and -0.68, respectively (*p* ≤ 0.035) (Fig. 6b, e), as well as the total number of astroglial cells, with *r* values of -0.63 and -0.64, respectively (*p* ≤ 0.044) (Fig. 6c, f).

## Discussion

While an increasing number of clinical investigations have highlighted the potential impact of the time intervals between rmTBI on the subsequent severity of outcomes [9, 35], the inherent diversity and heterogeneity within the realm of clinical mTBI may hinder the investigation of the pathophysiological change following cumulative injuries [36, 37]. Moreover, it is challenging to monitor chronic injury after rmTBI in clinical patients due to the high dropout rate, particularly beyond the initial year post-injury [38]. In the current study, we addressed this complexity by utilizing a meticulously controlled CHI animal model [27] showing long-term anxiety-like behavior after 50 days post-rmTBI (Supplementary Fig. S4c, d). We proposed the long-term alterations in DTI metrics occurring beneath the primary impact site and within remote WM regions following rmTBI. To the best of our understanding, our study reported changes in tensor scalar transformation metrics, encompassing *L* and *q* values, following rmTBI for the first time, highlighting the sensitivity of tensor scalar measurements to subtle injury in the remote WM. More importantly, a negative correlation was observed between *q* values and astroglial cells in the EC, reflecting the underlying mechanism of the alteration in the anisotropic component.

Our study observed reduced cortical FA from day 50 to day 90 in the animals after rmTBI with short intervals [39, 40]. In addition, we found decreased cortical *q* values in the animals at day 50 post-rmTBI with the short intervals. The *q* value, derived from tensor metrics decomposition, relied on the inherent distribution and orientation of water molecules, potentially indicative of tissue characteristics. This parameter has demonstrated effectiveness in distinguishing between gross and infiltrative tumor margins [22], low-grade and high-grade gliomas [24], and differentiating glioblastoma from metastases [33]. We postulate that substantial water retention in the cortex, resulting from neuronal loss and imbalanced microglial activity after injury [41], might reduce both FA and *q* values. Taken together, our findings suggest that in the chronic phase, rmTBI with shorter inter-injury intervals may lead to more pronounced neuronal loss and heightened neuroinflammatory response in the cortex compared to longer inter-injury intervals.

In contrast to Wallarian degeneration in moderate to severe injury showing the disruption of WM organization, elevated FA after mTBI has been reported in the chronic phase after concussive injuries [42, 43] postulated from cytotoxic edema and local inflammation with preserved water diffusion in WM [44–46]. Our finding, demonstrating significant local neuroinflammation but preserved myelination (Supplementary Fig. 3e), suggested that axonal swelling induced by WM stretching underneath the impact site may contribute to the increased FA and *q* values [44]. Our results were in line with previous research showing increased *q* values in WM under predominant stretch/compression due to normal pressure hydrocephalus [18]. The unchanged total magnitude of diffusion denoted by L from the compression of the extracellular space due to axonal swelling may further support our hypothesis. Moreover, it is also plausible that the increased FA may reflect the loss of crossing fibers or the compensatory alteration in the chronic injury. This complex interplay of factors highlights the multifaceted nature of FA alterations post-mTBI and the intricate relationship between microstructural changes and diffusion metrics.

Secondary brain injury in remote regions after TBI has been proposed to be associated with reactive gliosis in various brain areas, including the entorhinal cortex, hippocampus, or even the contralateral cortex and cerebellum, lasting for years [47]. However, little changes in DTI parameters [48, 49] or their recovery over time have been noted in the remote WM in various animal models of mTBI or rmTBI [30, 44], indicating that DTI measurement, particularly FA, may not be sensitive enough to correlate with neuropathological change induced by the secondary injury [29, 48]. In the current study, while no significant alterations in FA were observed in the remote WM, changes in *L* and *q* values were detected in the ipsilesional EC and IC after rmTBI. At day 90, lower *q* and *L* values, as well as lower MD (Supplementary Fig. S1b), were observed in the EC and IC. Given that FA represents the tissue anisotropic diffusion but is weighted by its total diffusion (L), FA would change only when there is a disproportionate alteration between *q* and *L* values [20, 50]. Consequently, the similar trend of *q* and *L* values in our results may contribute to the nonsignificant changes in FA values in the remote WM. Lower *L* values, indicating increased cellularity or vascularity, have been reported in the adjacent area surrounding high-grade gliomas [24]. The reduction of *q* values has been proposed to reflect the infiltrative environment of the peritumoral regions of GBM [30] and the loose arrangement of keratin flakes in the intracranial epidermoids [25], suggesting that *q* value may serve as an index for water diffusion in the extracellular environment. Together with our findings showing the overexpression of astrocytes and microglia but no significant loss of myelin in the EC and IC, the current study suggests that the lower *L* and *q* values may potentially reflect the astrogliosis in the remote WM with preserved axonal integrity after rmTBI. Our results, thus, may provide a potential index by using tensor decomposition to reflect the secondary injury in the WM with subtle neuronal structural disruption. Recently, increased orientation dispersion index, rather than changes in FA, in the optic tract of mice has been demonstrated to reflect the filtration of inflammatory cells after mTBI [51]. Future work combining multishell diffusion with biological modeling and immunohistology may further elucidate the specificity of different diffusion measures to cellular density and processes in the space surrounding the axons after rmTBI. Taken together, our results underscore the sensitivity of tensor scalar measurements in elucidating subtle changes in the remote WM in the chronic phase following rmTBI. Of note, no significant changes in any of the tensor scalar metrics were observed in the medial CC, further supporting that WM in the lateral brain may be more vulnerable to long-term inflammation after rmTBI.

In this study, several limitations had to be considered. First, our CHI model does not encompass the linear translation and rotation forces commonly encountered in human cases of mTBI. To better replicate the effects of acceleration-deceleration injuries in the brain, future research using unrestrained animals, such as a closed-head impact model of engineered rotational acceleration (CHIMERA) [52], will be needed. Second, while the changes of FA, *L*, and *q* values were observed at days 50 and 90 in our selected ROIs, these results may not be extrapolated to other time points following rmTBI or other brain regions. Future studies combining tract-based spatial statistics (TBSS) may provide a comprehensive WM map at the whole brain scale to illustrate the tentative changes following brain injury [30]. Finally, while our study highlights the sensitivity of tensor metrics in detecting the effects of rmTBI, the specificity of these changes to the underlying mechanisms remains to be studied. Despite not anticipating a significant correlation among individual tensor metrics based on previous studies [53, 54], a comprehensive multivariable analysis may clarify the alteration in these MRI features in relation to subtle microstructural abnormalities following rmTBI.

In summary, our results support the hypothesis that rmTBI with shorter inter-injury intervals is associated with more pronounced cerebral alteration, as evidenced by DTI metrics and neuropathological changes. These alterations are not confined to the parenchyma beneath the primary impact site but also affect remote WM in the ipsilesional hemisphere. Notably, the WM in the lateral part of the brain exhibits greater susceptibility to chronic injury. Furthermore, our results reveal that the anisotropy component, *q* value, is sensitive to astrogliosis surrounding the subtle axonal damage, highlighting its potential for quantifying remote WM changes following rmTBI.

### Supplementary information

**Additional file 1: Supplementary Table. 1.** 7-Tesla MRI parameters. **Supplementary Table. 2.** ROI information. **Supplementary Table. 3.** IHC antibody. **Supplementary Figure. 1.** Mean MD from the cortex, corpus callosum (CC), and hippocampus (HPC) under the impact site (a), and the medial corpus callosum (medial CC), external capsule (EC), and internal capsule (IC) (b) at day 50 and day 90 in the sham group and after rmTBI. * indicates significant among groups (*p* > .05). **Supplementary Figure. 2.** Regions of interest (ROIs) of the cortex, corpus callosum (CC), and hippocampus (HPC) under the impact site, and the medial corpus callosum (medial CC), external capsule (EC), and internal capsule (IC) outline in the immunohistochemistry images of NeuN (a), GFAP (b), CD68 (c), and MBP (d) for quantitative analysis. (e) MBP staining of WM at 90 days after rmTBI. **Supplementary Figure. 3.** Correlation of the total diffusion magnitude (L value) in the EC (a-c) and IC (d-f) at day 90 after rmTBI with the corresponding IHC-positive cells. The white, gray, and black dots denoted data from the sham, short-, and long-interval groups. **Supplementary Figure. 4.** Move duration (a), travel distance (b), center entries (c), and center time (d) at days 50 and 90 after rmTBI. Significant lower Center Entries were shown in the long interval group at days 50 and 90 after rmTBI. Significant higher Center Time was shown in the short interval group at days 50 and 90 after rCHI. * indicates significant among groups ((*p* >.05).

## Data Availability

The datasets used and/or analyzed during the current study are available from the corresponding author upon reasonable request.

## Abbreviations

CC: Corpus callosum
CD-68: Cluster of differentiation 68
CHI: Closed-head injury
DTI: Diffusion tensor imaging
EC: External capsule
FA: Fractional anisotropy
GFAP: Glial fibrillary acidic protein
IC: Internal capsule
L: Total value of diffusion tensor
MD: Mean diffusivity
MRI: Magnetic resonance imaging
mTBI: Mild traumatic brain injury
NeuN: Neuronal nuclear antigen
q: Anisotropic component of diffusion tensor
rmTBI: Repetitive mTBI
ROI: Region of interest
WM: White matter
