# Supplementary material for: Anisotropy component of DTI reveals long-term neuroinflammation following repetitive mild traumatic brain injury in rats
Source: Eur Radiol Exp. 2024 Jul 24;8:82. doi: 10.1186/s41747-024-00490-w (PMC11269550; doi:10.1186/s41747-024-00490-w)
Supplement: Supplementary file 1 — Additional file 1: Supplementary Table. 1. 7-Tesla MRI parameters. Supplementary Table. 2. ROI information. Supplementary Table. 3. IHC antibody. Supplementary Figure. 1. Mean MD from the cortex, corpus callosum (CC), and hippocampus (HPC) under the impact site (a), and the medial corpus callosum (medial CC), external capsule (EC), and internal capsule (IC) (b) at day 50 and day 90 in the sham group and after rmTBI. * indicates significant among groups (p > .05). Supplementary Figure. 2. Regions of interest (ROIs) of the cortex, corpus callosum (CC), and hippocampus (HPC) under the impact site, and the medial corpus callosum (medial CC), external capsule (EC), and internal capsule (IC) outline in the immunohistochemistry images of NeuN (a), GFAP (b), CD68 (c), and MBP (d) for quantitative analysis. (e) MBP staining of WM at 90 days after rmTBI. Supplementary Figure. 3. Correlation of the total diffusion magnitude (L value) in the EC (a-c) and IC (d-f) at day 90 after rmTBI with the corresponding IHC-positive cells. The white, gray, and black dots denoted data from the sham, short-, and long-interval groups. Supplementary Figure. 4. Move duration (a), travel distance (b), center entries (c), and center time (d) at days 50 and 90 after rmTBI. Significant lower Center Entries were shown in the long interval group at days 50 and 90 after rmTBI. Significant higher Center Time was shown in the short interval group at days 50 and 90 after rCHI. * indicates significant among groups ((p >.05). [file 41747_2024_490_MOESM1_ESM.pdf]

Anisotropy component of DTI reveals long-term neuroinflammation following repetitive mild traumatic brain injury in rats

ELECTRONIC SUPPLEMENTARY MATERIAL

SUPPLEMENTARY TABLE 1

7-Tesla MRI parameters

	**T2-weighted (TurboRARE)**	**DTI (4-shot SE-EPI)**
**Orientation**	Coronal	Coronal
**Acquisition matrix**	256 x 256	96 x 96
**Field of view (cm)**	2.0 x 2.0	2.0 x 2.0
**Echo train length**	8	-
**Repetition time (ms)**	3,600	3,000
**Echo time (ms)**	40	28
**Number of slices**	16	16
**Slice thickness (mm)**	1	1
**Others**	--	$\delta/\triangle$ = 5/15 ms, number of B0 = 5, b-value = 1000 s/mm^2 , number of directions = 30
**Acquisition time**	7 min 40 s	14 min
**Average**	4	2

SUPPLEMENTARY TABLE 2

ROI information

Region of interest (ROI)	Cortex	CC	HPC	Medial CC	EC	IC
**Shape**	Rectangular ROIs			Manually delineated ROIs		
**Size**	1.5 x 2.5 (mm^2 per slice)	0.5 x 2.5 (mm^2 per slice)	1 x 2.5 (mm^2 per slice)	2.5 (mm^3)	3.0 (mm^3)	4.5 (mm^3)
**Coordinates (mm posterior to the Bregma)**	1.5-3.5	1.5-3.5	1.5-2.5	1.5-3.5	1.5-3.5	1.5-3.5
**Number of image slices**	3	3	2	3	3	3
**Based MR image**	T2-weighted images			FA maps with threshold FA > 0.35		

SUPPLEMENTARY TABLE 3

IHC antibody

**Targets**	**neurons**	**astrocytes**	**microglia**	**mature myelin and the structure of myelin sheath**
**Antibody**	hexaribonucleotide binding protein-3 (NeuN)	anti-glial fibrillary acidic protein (GFAP)	anti-Cluster of Differentiation 68 (CD 68)	myelin basic protein (MBP)
**Vendor**	Millipore	Bioworld	BioSB	Bioworld
**Cat.**	MAB377	BS6460	BSB5292	BS90829

SUPPLEMENTARY FIGURE 1

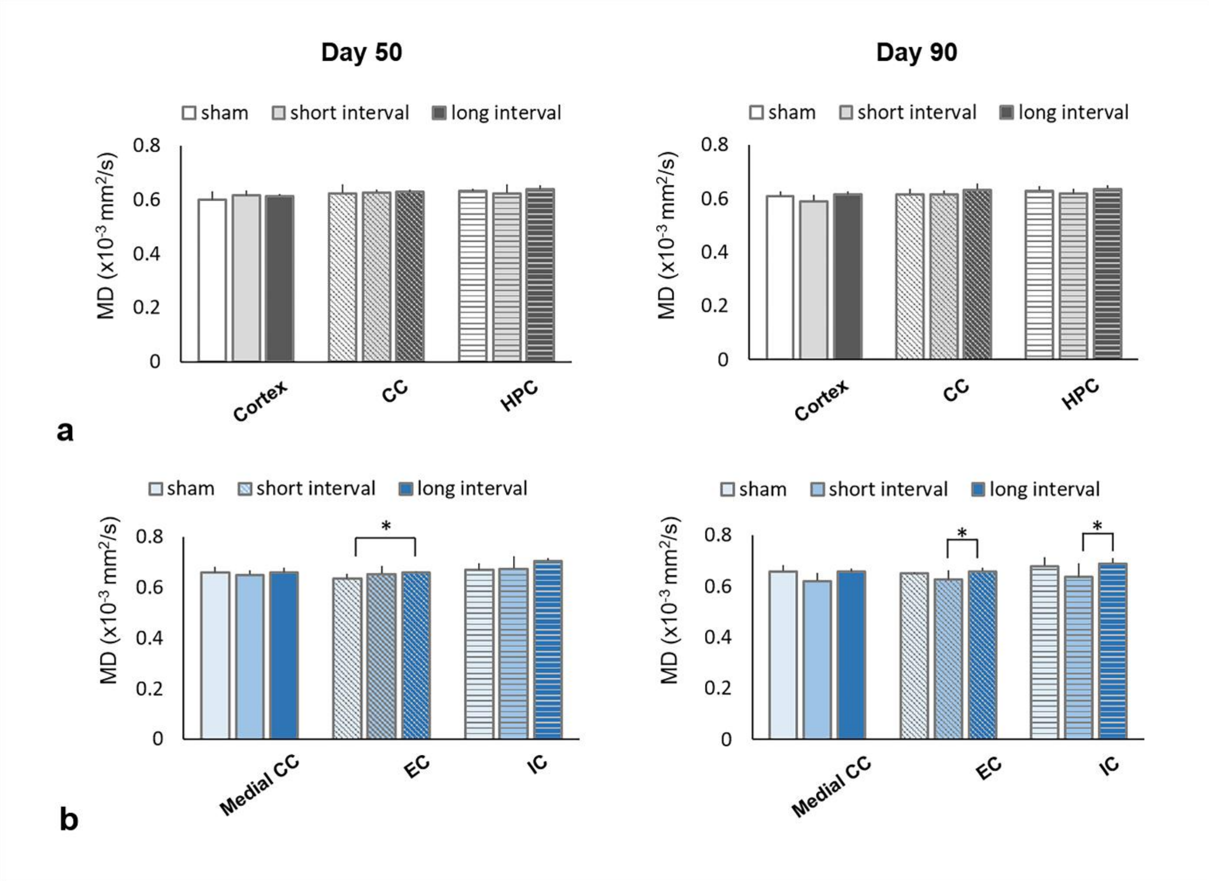

Supplementary Figure 1. Mean MD from the cortex, corpus callosum (CC), and hippocampus (HPC) under the impact site (a), and the medial corpus callosum (medial CC), external capsule (EC), and internal capsule (IC) (b) at day 50 and day 90 in the sham group and after rmTBI. * indicates significant among groups ($p < .05$).

SUPPLEMENTARY FIGURE 2

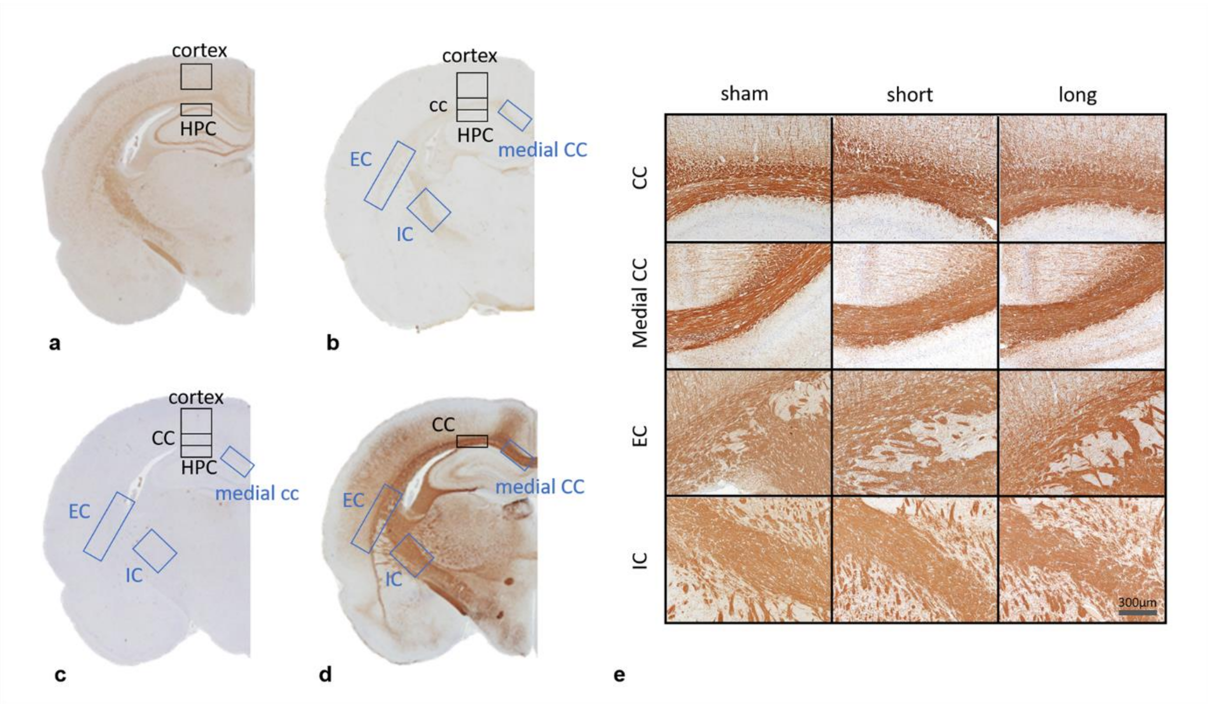

Supplementary Figure 2. Regions of interest (ROIs) of the cortex, corpus callosum (CC), and hippocampus (HPC) under the impact site, and the medial corpus callosum (medial CC), external capsule (EC), and internal capsule (IC) outline in the immunohistochemistry images of NeuN (a), GFAP (b), CD68 (c), and MBP (d) for quantitative analysis. (e) MBP staining of WM at 90 days after rmTBI.

SUPPLEMENTARY FIGURE 3

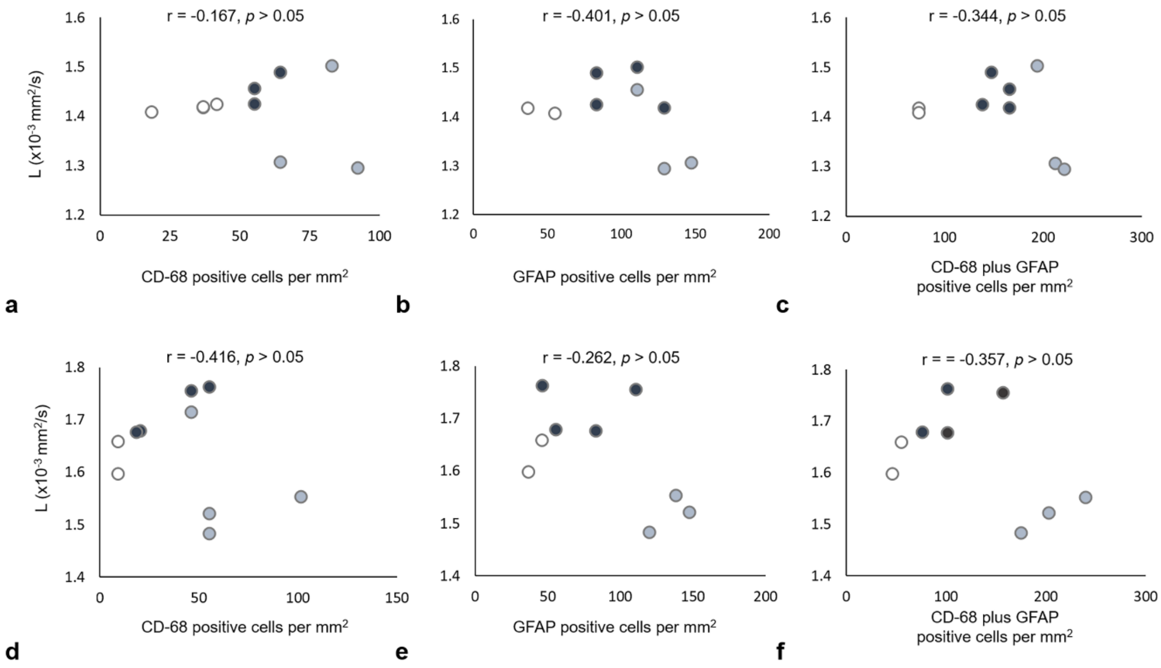

Supplementary Figure 3. Correlation of the total diffusion magnitude (L value) in the EC (a-c) and IC (d-f) at day 90 after rmTBI with the corresponding IHC-positive cells. The white, gray, and black dots denoted data from the sham, short-, and long-interval groups.

SUPPLEMENTARY FIGURE 4

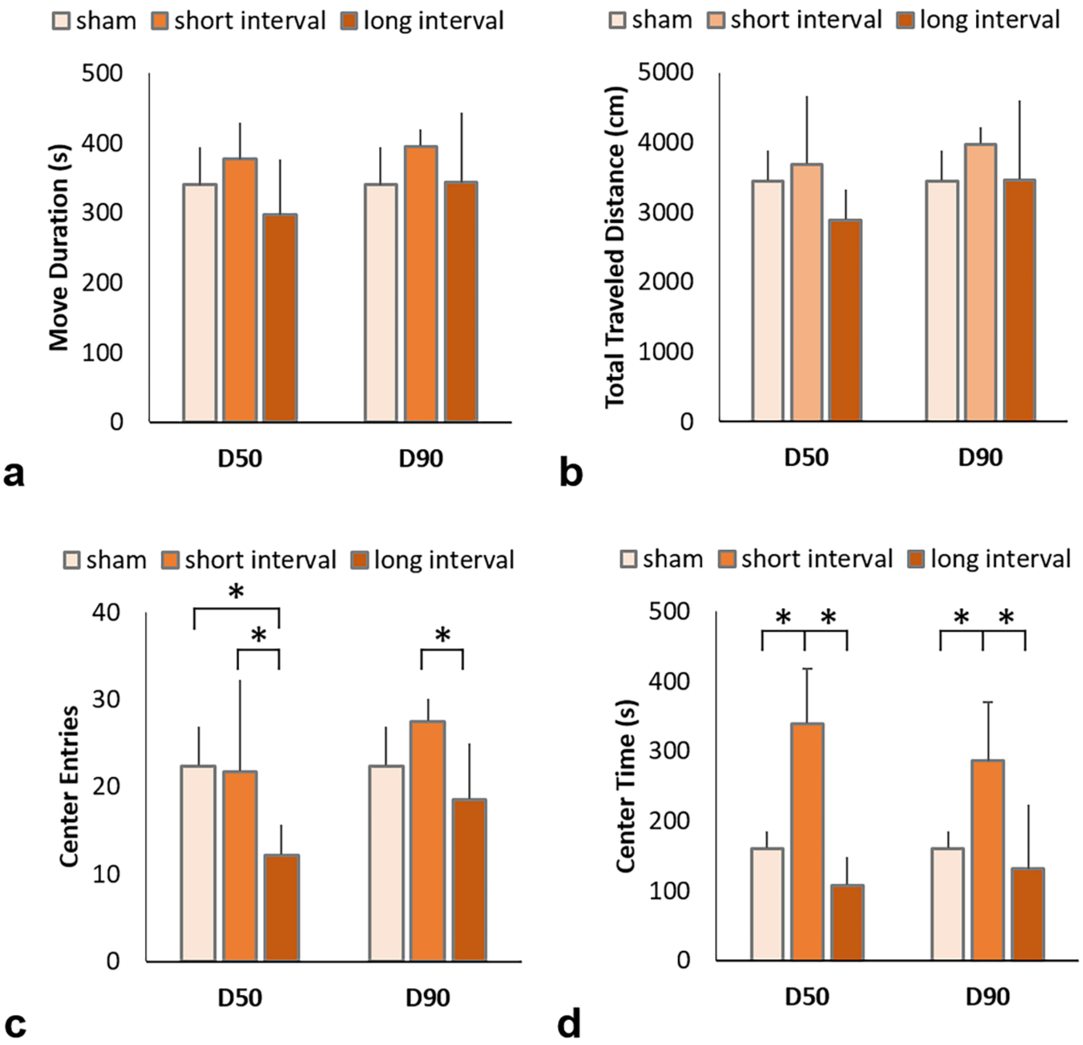

Supplementary Figure 4. Move duration (a), travel distance (b), center entries (c), and center time (d) at days 50 and 90 after rmTBI. Significant lower Center Entries were shown in the long interval group at days 50 and 90 after rmTBI. Significant higher Center Time was shown in the short interval group at days 50 and 90 after rCHI. * indicates significant among groups ($p < .05$).

SUPPLEMENTARY TEXT

Behavioral assessment

The open-field test was used to assess spontaneous activity and anxiety-like behavior at 50 and 90 days after rmTBI. The open-field setting was composed of the open arena (50 × 35 cm) and the center region, which was defined as the inner rectangular zone (40 × 25 cm) at the center [1]. During the 10-minute test, animals were monitored using a video camera. Tracking and calculating the movement duration, mean travel distance, center entries, and center time of each animal during the trial were analyzed using Smart 3.0 (Panlab, Barcelona, Spain) [2; 3]. The configuration setting used in the current study was adjusted based on the manual of Smart 3.0. The software automatically calculated the moving duration, total distance, center entries, and center times for each trail.

1 Kao YJ, Lui YW, Lu CF, Chen HL, Hsieh BY, Chen CY (2019) Behavioral and Structural Effects of Single and Repeat Closed-Head Injury. AJNR Am J Neuroradiol 40:601-608

2 Izumi H, Sato K, Kojima K, Saito T, Saido TC, Fukunaga K (2020) Oral glutathione administration inhibits the oxidative stress and the inflammatory responses in App(NL-G-F/NL-G-F) knock-in mice. Neuropharmacology 168:108026

3 Llorca-Torralba M, Pilar-Cuellar F, da Silva Borges G, Mico JA, Berrocoso E (2020) Opioid receptors mRNAs expression and opioids agonist-dependent G-protein activation in the rat brain following neuropathy. Prog Neuropsychopharmacol Biol Psychiatry 99:109857
